# Rising role of prescription drugs as a portal to injection drug use and associated mortality in Baltimore, Maryland

**DOI:** 10.1371/journal.pone.0213357

**Published:** 2019-03-04

**Authors:** Javier A. Cepeda, Jacquie Astemborski, Gregory D. Kirk, David D. Celentano, David L. Thomas, Shruti H. Mehta

**Affiliations:** 1 Department of Medicine, University of California San Diego, La Jolla, California, United States of America; 2 Department of Epidemiology, Johns Hopkins Bloomberg School of Public Health, Baltimore, Maryland, United States of America; 3 Division of Infectious Diseases, Johns Hopkins School of Medicine, Baltimore, Maryland, United States of America; RTI International, UNITED STATES

## Abstract

**Introduction:**

Prescription drug abuse is a major public health problem in rural and suburban areas of the United States, however its emergence in large urban settings with endemic injection drug use remains understudied. We examined temporal trends in injection drug use initiation and mortality among people who inject drugs (PWID) in Baltimore, Maryland.

**Methods:**

Data were derived from the baseline assessment of PWID enrolled in a community-based cohort study with longitudinal follow-up for mortality assessment. PWID were recruited from 2005–2008 (N = 1,008) and 2015–2018 (N = 737). We compared characteristics by birth cohort (before/after 1980) and type of drug initiated (prescription opioids, prescription non-opioids, non-injection illicit drugs, or injection drugs). We calculated standardized mortality ratios (SMR) using the US general population as the reference.

**Results:**

PWID born after 1980 were more likely to initiate drug use with prescription opioids and non-opioids and had higher levels of polysubstance prior to injection initiation, compared to individuals born before 1980. Overall mortality was high: 2.59 per 100 person-years (95% CI: 2.27–2.95 per 100 person-years). Compared to the US population, the highest SMRs were observed among participants between 40–44 years of age, with especially high mortality among women in this age group (SMR:29.89, 95% CI: 15.24–44.54).

**Conclusions:**

Mirroring national trends, the profile of PWID in Baltimore has changed with increased prescription drug abuse and high levels of polysubstance use among younger PWID. Interventions need to reach those using prescription drugs early after initiation of use in order to reduce transition to injecting. Urgent attention is warranted to address premature mortality, particularly among middle-aged and female PWID.

## Introduction

Non-medical prescription drug use, particularly misuse of prescription opioids, is a major and growing public health crisis in the United States. Illicit and non-medical opioid use can greatly heighten the risk of fatal overdose as evidenced by the rise in the number of opioid-related deaths in the U.S. In 2016 an estimated 59,000–65,000 deaths in the U.S. were attributed to drug overdose [1] and it has recently become the most common cause of death among Americans under 50 years of age. Powerful synthetic opioids, such as fentanyl mixed with heroin, have been implicated as a reason for the more recent surge in drug overdose related deaths [2, 3].

The consequences of prescription drug use are most pronounced among those transitioning from non-injection prescription drug use (often licit) to injection of prescription drugs and/or heroin [4–10]. In multiple settings, prescription opioid dependence has been significantly associated with transition to heroin use, [11] with an eventual transition to injection [12]. Moreover, some findings have suggested that this transition may be more rapid among younger users. For example, in a study among people who inject drugs (PWID) from California, individuals born in the 1980s/1990s demonstrated a more rapid transition to injection drug use than PWID born in the 1970s [13]. Associations between use of other non-opioid prescription drugs (e.g., sedatives and tranquilizers), which are prevalent in some groups, and transitions to injection are less clear. For example, data from a cohort of people who inject drugs in rural Kentucky indicated that prescription sedatives were associated with a 2.5-fold increased risk of injection initiation [10]. Similarly, tranquilizer/sedative use was found to be associated with a 3.3 fold increased risk of injection initiation among street youth in Montreal [14], however this association was not retained in the multivariable model. The public health implications of this transition to injection drug use are substantial since injection drug use can facilitate transmission of blood-borne diseases, such as HIV and hepatitis C virus (HCV). Indeed, sharing injection equipment for recreational use of the opioid analgesic oxymorphone fueled a recent HIV outbreak in rural Southeastern Indiana [15] as well as an increase in the number of new HCV cases from 2006–2012 in four Appalachian states [16].

This transition from non-injection prescription drug use, whether opioid or non-opioid, to injection use also represents a critical opportunity for interventions to engage users in key harm reduction and other preventive services. However, in many large urban centers such as Baltimore, the pathway to injection drug use has historically been through the use of non-injection cocaine and heroin [17, 18]. Understanding how this pathway has changed will be critical for designing interventions to prevent the transition to and minimize the harms associated with injection drug use. Indeed, the most consequential of these harms is mortality. Specifically, injection drug use can be a direct driver of mortality (e.g. drug overdose [19], skin and soft tissue infections, [20] infective endocarditis, and blood-borne viruses such as HIV and HCV [21]) or indirect driver (e.g. criminal justice involvement [22], poor access to preventive care [23], violence [24]).

Nonetheless, due to the emergence fentanyl and other synthetic opioids in the illicit drug market, mortality risk due to drug overdose is still of primary importance. At the state level, Maryland has experienced one of the most precipitous increases in overdose-related deaths, rising from 21 deaths per 100,000 in 2015 to 36 deaths per 100,000 in 2016 [25]. Baltimore, the largest city in Maryland, has one of the highest per capita rates of injection drug use and one of the highest overdose mortality rates in the country [26–28]. It has been estimated that among the 621,000 inhabitants of Baltimore, nearly 25,000 individuals have opioid use disorder, 20,000 of whom regularly use heroin [29]. Injection drug use has historically been concentrated in East and West Baltimore City but recent reports suggest shifts outside of this area with increased injection drug use in suburban Baltimore County. Given the changing patterns of drug use and mortality at the national level, we investigated whether these shifts were also being observed among participants from a large urban drug using cohort that has been ongoing since 1988. Specifically, our objectives were to 1) characterize temporal trends in trajectories of drug use initiation and transitions to injection by birth cohort; and 2) compare overall trends in mortality and among those who initiated drug use through different pathways and by birth cohort in a community-based sample of PWID participating in a longitudinal research study in Baltimore, Maryland.

## Methods

### Study participants

Data originated from the ALIVE (AIDS Linked to the IntraVenous Experience) study, a prospective, community-based cohort of former and current PWID in Baltimore, Maryland [30]. Enrollment began in 1988 and included 2,938 persons. Since then, additional participants have been enrolled in 1994–95 (n = 434), 1998 (n = 295), 2005–2008 (n = 1,008) and 2015–2018 (n = 737). Eligibility criteria for participating in the study include being at least 18 years of age and a history of injecting drugs in the prior 10 years. We recruited participants by placing flyers at drug treatment programs, syringe service programs, community health and HIV clinics, health fairs, and other community outreach activities. Individuals could also be referred by word of mouth from participants already enrolled in the study. This analysis was restricted to the two most recent recruitment periods (2005–2008 and 2015–2018) when participants were specifically asked about prescription drug use, route of administration (e.g., swallowed, snorted, smoked, injected), and the age at which they first consumed each type of drug. Recruitment and enrollment procedures did not change between the 2005–2008 and 2015–2018 recruitment periods. The Johns Hopkins University Institutional Review Board approved the study protocol and all study participants provided written informed consent.

### Measurements

At baseline, participants were asked about lifetime medical history, risk behaviors, and age at initiation of individual drugs, using an interviewer-administered questionnaire. Geographical residence (Baltimore City versus County) of participants was determined by geocoding the zip code where they received their mail. All participants were tested for HIV and for HCV antibodies at baseline.

Our independent variable of interest was self-reported first drug used. Participants were classified into five groups based on the minimum age of the first drug they used. The first group consisted of individuals who initiated with non-medical prescription opioids. The second group included individuals who initiated with non-medical prescription drugs not considered to be opioids (i.e., non-opioids that were primarily sedatives and tranquilizers). The third group was comprised of individuals who used “traditional” non-injection illicit drugs (primarily cocaine and heroin) prior to injection that have been previously documented in Baltimore as a pathway to injection drug use [17, 18]. In some instances, participants reported using prescription drugs (either opioid or non-opioid) and non-injection illicit drugs at the same age. Because we could not determine temporality, these participants were classified into a fourth group. Lastly, the fifth group consisted of participants who reported injecting drugs at the same age (or before) as prescription drugs and/or non-injection illicit drugs. We did not consider marijuana in our drug initiation classifications since over 80% used it as the first illicit drug. Additionally, the association between marijuana and progression to other illicit drugs has been well researched [31] and its inclusion as a separate drug initiation group would limit insight on the role of emergent non-medical prescription drug use as a potential pathway to injection versus traditional illicit drugs.

There were differences between the 2005–2008 and the 2015–2018 surveys with respect to inquiry on prescription drug use. The 2005–2008 survey included items that asked specifically about oral, non-medical use of prescription opioids (oxycontin, percocet, buprenorphine and methadone) as well as prescription non-opioids (benzodiazepines and clonidine). The 2015–2018 elicited information on broader prescription drug classes, including opioids (methadone, oxycontin, percocet, codeine, darvon, percodan, dilaudid, demerol, buprenorphine), sedatives (sleeping pills, barbiturates, seconal, quaaludes, chloral hydrates, clonidine), tranquilizers or anti-anxiety (valium, Librium, muscle relaxant, benzodiazapines, Klonopin, Valium, Ativan, Xanax), and stimulants (preludin, benzedrine, methedrine, uppers, speed, Ritalin, Dexedrine, Adderall). Sedatives, tranquilizers, and stimulants were classified as prescription non-opioids and separated from prescription opioids due to inconsistent evidence on their association with injection initiation [10, 14].

We also characterized polysubstance use before transition to injection for both recruitment cohorts. We summed the number of drugs that were used via a non-injection route prior to when the participants indicated that they began injecting drugs. Individuals who reported injecting drugs at the same age as using other non-injection drugs were excluded from the polysubstance use analysis.

### Mortality

Mortality data were obtained from the National Death Index (NDI) with confirmation from death certificates. Persons were censored at their date of death or December 2016 (through which NDI data were complete). Thus, only individuals recruited before January 2017 were included in this analysis.

### Statistical analyses

All analyses are presented stratified by recruitment cohort given the differences in the surveys over time. We characterized the proportion who initiated drug use by different drug type/route and the decade of their birth (birth cohort). Descriptive statistics were used to characterize the sociodemographics and risk behaviors by three groups defined by the first drug used as described above. Chi-square and Wilcoxon rank sum tests were used to determine differences between the two recruitment periods for categorical and continuous variables respectively. We used the Cochran-Armitage test to detect temporal trends. Crude mortality rates (MR) were expressed per 100 person-years (PY), which were calculated using the person-time method [32]. Age and sex- standardized mortality ratios (SMR) were calculated by dividing the observed number of deaths by the expected number of deaths using the 2010 U.S. population as the reference [33, 34]. All statistical analyses were conducted using SAS version 9.4 (SAS Institute, Cary, North Carolina).

## Results

### Sample characteristics by recruitment period

Overall, 57% (578/1008) of participants in 2005–08 initiated drug use with non-injection drugs as compared to 72% (529/737) participants in 2015–18. Among these participants, approximately 15% of participants reported residing outside of Baltimore City, however there was a shift over time in the demographic characteristics, with increasing proportions of male (65% vs. 72%) and non-African American participants (34% vs. 45%) (Table 1). We also found an increasing trend in the proportion of participants who initiated with prescription drugs (either opioid or non-opioid), with or without other illicit non-injection drugs (16% vs. 28%).

**Table 1:** Characteristics by recruitment period of PWID in Baltimore.

	2005–08 recruitment period (N = 1008)	2015–18 recruitment period (N = 737)
**Sociodemographics**		
Male	654 (65)	531 (72)
Geographical residence		
City	831 (85)	596 (84)
County	147 (15)	116 (16)
Age (median, IQR)	44 (36–49)	46 (36–53)
Birth decade		
Before 1960	315 (31)	98 (13)
1960–1969	403 (40)	253 (34)
1970–1979	201 (20)	188 (26)
1980 or later	89 (9)	198 (27)
Race/ethnicity		
African American	671 (67)	406 (55)
White	298 (30)	294 (40)
Other	39 (4)	37 (5)
At least high school education	432 (43)	377 (51)
**Substance use and risk behaviors**		
Doctor ever diagnosed with alcohol use disorder	185 (19	135 (18)
Drug initiation (type of drug used)		
Prescription opioids	61 (6)	25 (3)
Prescription non-opioids	64 (6)	87 (12)
Prescription drugs*/non-injection illicit drugs	44 (4)	94 (13)
Non-injection illicit drugs	407 (40)	323 (44)
Injection drugs	432 (43)	208 (28)
Frequency of injection drug use		
3x/day for ≥ 3 days	542 (54)	503 (68)
1-2x/day for ≥ 3 days	190 (19)	121 (16)
Daily	114 (11)	33 (5)
4-6X/week	74 (7)	25 (3)
2-3X/week or less frequent	88 (9)	54 (7)
Median number of years between non-injection and injection drug use	5 (2–9)	6 (3–11)
Ever use needle after someone else	758 (75)	461 (63)
Ever pass needle	752 (75)	490 (67)
Ever binge injection drug use	876 (87)	608 (84)
Ever visit shooting gallery	863 (86)	602 (82)
Ever been in jail for more than 7 days	870 (86)	668 (91)
Ever overdosed	610 (61)	517 (70)

*either prescription opioid or non-opioid

### Drug use initiation and transitions to injection by birth cohort

**Fig 1:**
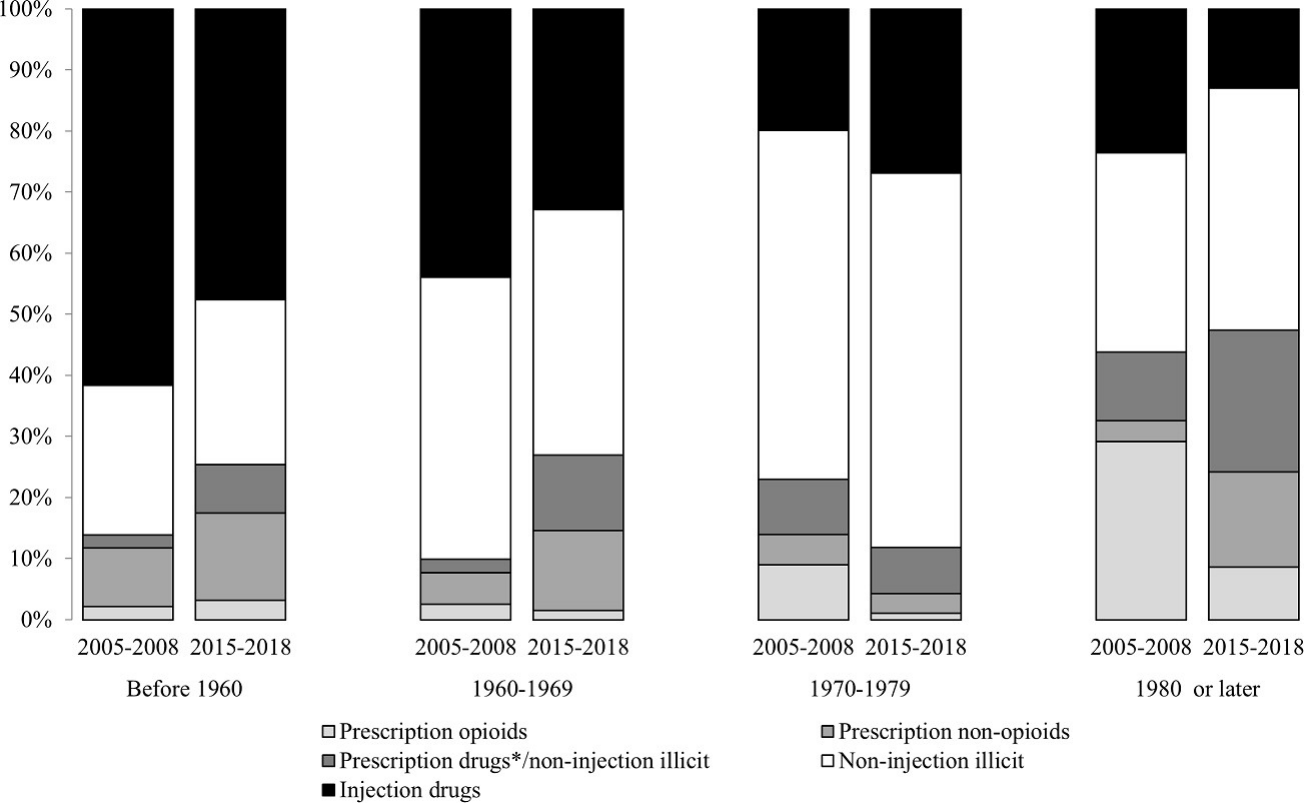
Temporal trends in drug use initiation by birth and recruitment period. The figure displays the type of drug first used (prescription opioids, prescription non-opioids, prescription drugs and non-injection illicit drugs, and non-injection illicit drugs) by birth cohort (Before 1960, 1960–69, 1970–79, 1980 or later) and recruitment into the ALIVE study (2005–08 and 2015–2018). Test for trend across birth cohort for both recruitment period p<0.001. *Prescription drugs includes both opioid and non-opioid.

We further compared patterns of drug use initiation by birth cohort separately within each recruitment period. As shown in Fig 1, the proportion of individuals initiating use with prescription drugs (either opioid or non-opioid), has been increasing by birth decade (p<0.001). For example, in the 2005–2008 recruitment period, 2% of individuals born before 1960 initiated with prescription opioids, compared to 29% among individuals born in 1980 or later. Similar trends were observed in the 2015–2018 recruitment period, where 25% of individuals born before 1960 initiated with prescription drugs, increasing to 45% among those born after 1980. Consistent across both recruitment periods, initiation by drug type was significantly associated with geographical residence. Among participants who initiated with prescription drugs (either opioid or non-opioid), 25% lived outside of Baltimore City. By comparison, approximately 15% of participants who initiated with non-injection illicit and 10% with injection drugs lived outside of the city.

Moreover, compared to participants born prior to 1980, those born after 1980 tended to initiate drug use at younger ages regardless of which drug was used first (Table 2). This difference was fairly consistent across the two recruitment periods. In general, the transition to injection drug use was shorter for persons born after 1980 compared to those born before 1980. For example. in the 2005–2008 recruitment period, the median time to injection for those who initiated with prescription opioids was 5 years (IQR: 2–10) for those born prior to 1980 and 3.5 years (IQR: 2–5) for those born after 1980 (p = 0.09). Notable exceptions were participants recruited from 2015–2018 that initiated with prescription opioids or non-opioids. Those born after 1980 who initiated with illicit prescription opioids or non-opioids tended to have a longer median duration to injection drug use compared to their older counterparts, however these differences were not statistically significant.

**Table 2:** Patterns of drug use and median age of initiation and median years to injection by birth cohort and recruitment period.

	2005–2008 recruitment period		2015–2018 recruitment period	
	**Median age of illicit drug initiation (IQR)**			
	Born before 1980 **(n = 919)**	Born 1980 or later **(n = 89)**	Born before 1980 **(n = 539)**	Born 1980 or later **(N = 198)**
Prescription opioids	**17 (15–18)**	15 (14–17)^**^	16 (12–25)	16 (13–17)
Prescription non-opioids	17 (16–18)	15 (11–16)	**16 (14–18)**	14 (11.5–17)^*^
Prescription drugs^a^/non-injection illicit drugs	18 (16–20)	16 (15–17)	16 (15–18)	16 (15–17)
Non-injection illicit drugs	**18 (16–21)**	15 (13–16)^***^	**17 (15–19)**	16 (15–18)^**^
Injection only^b^	**18 (16–22)**	15 (14–16)^***^	**18 (16–23)**	16.5 (15–19)^*^
	**Median years to injection initiation (IQR)**			
	Born before 1980 **(n = 508)**	Born 1980 or later **(n = 68)**	Born before 1980 **(n = 357)**	Born 1980 or later **(n = 172)**
Prescription opioids	5 (2–10)	3.5 (2–5)	5 (3–13)	6 (2–7)
Prescription non-opioids	6 (2–10)	4 (2–4)	7 (3–13)	8 (4–10)
Prescription drugs^a^/non-injection illicit drugs	4 (2–7)	2 (1–4)	5 (3–12)	4 (3–7)
Non-injection illicit drugs	**5 (2–10)**	3 (1–5)^**^	**7 (4–13)**	6 (3–9.5)^*^
	**Median number of drugs used prior to injection initiation (IQR)**			
	Born before 1980 **(n = 504)**	Born 1980 or later **(n = 68)**	Born before 1980 **(n = 348)**	Born 1980 or later **(N = 160)**
Prescription opioids	**2 (1–4)**	5 (3–6)^**^	3 (2–4)	5 (1–7)
Prescription non-opioids	2 (1–3)	3 (2–7)	**3 (2–5)**	5 (4–7.5)^**^
Prescription drugs^a^/non-injection illicit drugs	4 (3–5)	4 (2–6)	4 (3–6)	6 (4–7)
Non-injection illicit drugs	**2 (1–3)**	3 (2–6)^***^	**3 (2–4)**	5 (2–6.5)^***^

a. either prescription opioid or non-opioid

b. Median age to first injection drug use since injection drug use preceded or occurred at the same age as non-injection drugs in this group

Boldface indicates statistical significance difference within each recruitment period

*p<0.05

** p<0.01

*** p<0.001

Polysubstance use prior to injection initiation was common across all groups, but it was consistently higher among those born in 1980 or later. Among individuals who initiated with prescription opioids in the 2005–2008 recruitment period, the median number of drugs used prior to injection drug initiation was significantly higher among those born after 1980 (median: 5, IQR: 3–6) compared to those born before 1980 (median: 2, IQR: 1–4, p-value<0.001). Similar trends were observed among individuals who initiated with prescription opioids who were recruited from 2015–2018 born after 1980 (median: 5, IQR: 1–7) compared to those before 1980 (median: 3, IQR: 2–4), however this difference did not retain statistical significance. Among those who initiated with prescription non-opioids, polysubstance use prior to injection was significantly higher among those born in 1980 or later (p<0.01).

### All-cause mortality

**Fig 2:**
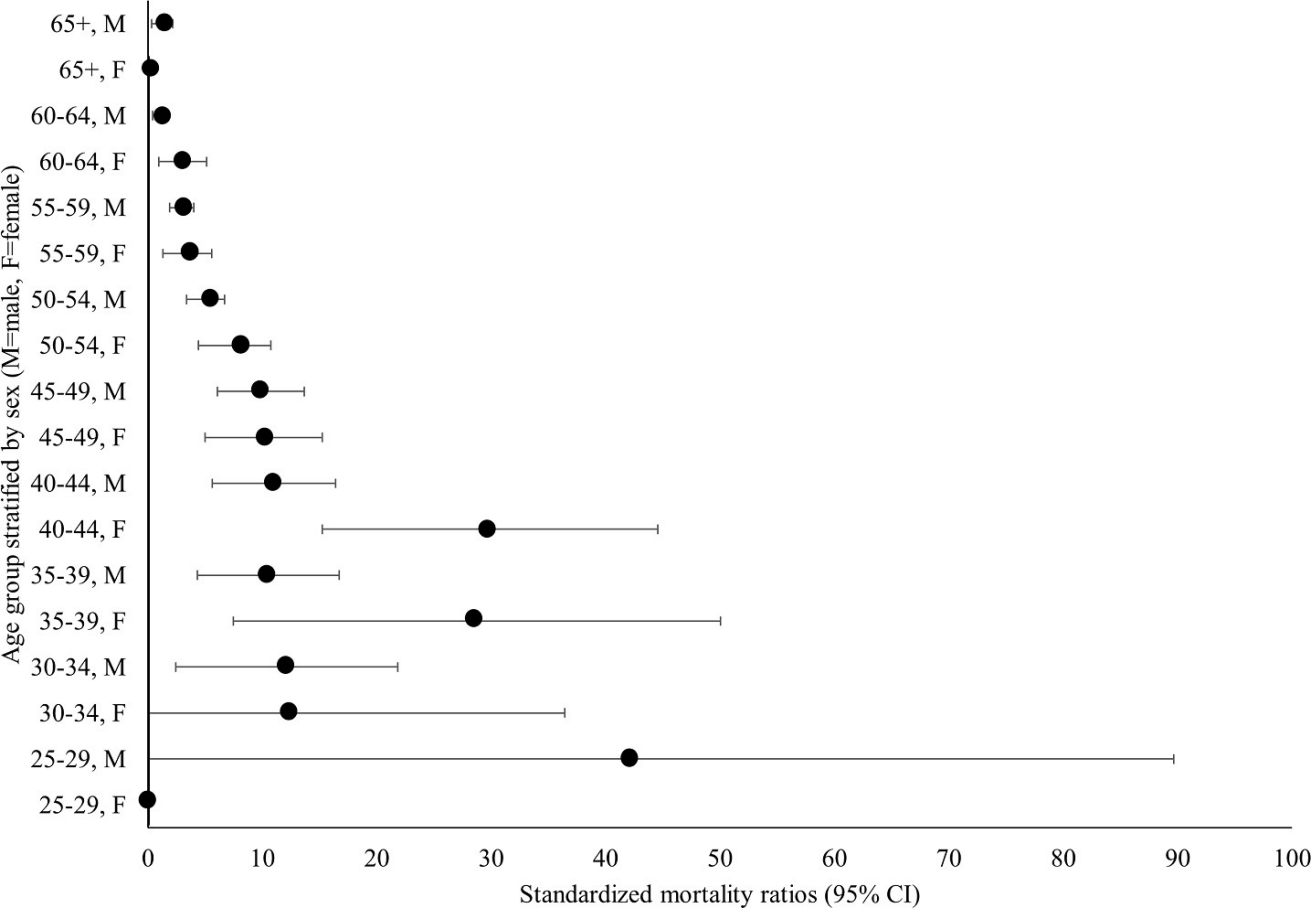
Standardized mortality ratios for PWID in the ALIVE study, stratified by age and sex. No deaths observed among females in 25–29 and >65 age groups.

Across both recruitment cohorts there were 226 deaths in 8,709 PY of follow-up through 2016 (mortality rate [MR]: 2.59 deaths per 100 PY, 95% CI: 2.27–2.95). Overall, the highest MR was observed among those who were recruited in the 2005–2008 period and had initiated with injection drugs (MR: 3.35 deaths per 100 PY, 95% CI: 2.79–4.02). However, mortality rates among those who initiated with prescription drugs (MR: 1.76, 95% CI: 1.12–2.76) and non-injection drugs (MR: 2.07 deaths per 100 PY, 95% CI: 1.65–2.60) were similar (see S1 File.). Among those born after 1980, the mortality rate among people who initiated with prescription opioids (1.37 per 100 PY) and prescription non-opioids (2.51 per 100 PY) was higher than those who initiated with non-injection (1.09 deaths per 100 PY), or injection drugs (1.10 deaths per 100 PY), however these rates were not significantly different. Age and sex specific SMRs are shown in Fig 2. The overall SMR was 4.43 (95% CI: 3.85–5.01). Compared to individuals in the general population who were born in the 1980s, mortality was 13-fold higher among individuals born in the 1980s in the ALIVE cohort (SMR: 13.26, 95% CI: 5.04–21.47). Additionally, compared to individuals in the general population born before 1980. Some of the highest SMRs were observed among participants aged between 35–39 and 40–44. For example, compared to similar age and sex specific groups in the general U.S. population, mortality was nearly 30-fold higher among females (SMR: 29.89, 95% CI: 15.24–44.54) and 11-fold higher among males (SMR: 11.02: 95% CI: 5.62–16.41) in the 40–44 age group.

## Discussion

Since 2000, Baltimore has experienced increased morbidity and mortality attributable to non-medical prescription drug use with a shift in the sociodemographic profile of PWID that mirrors national trends [35]. Until 2000, the demographic composition of this community-based cohort was consistently 85% African-American and fewer than 3% of the participants lived outside Baltimore City. However, over the past 18 years, our cohort has become more balanced with respect to race, which is consistent with national sociodemographic trends indicating that heroin use has been increasing among non-Hispanic Whites [36]. Moreover, the proportion of participants from the surrounding suburban counties has increased from 3% to more than 15%, despite no changes in our recruitment procedures. This sociodemographic shift has important implications as we found that among people who initiated with prescription drugs (either opioid or non-opioid), approximately 25% resided outside Baltimore City. Additionally, our study demonstrated how the pathways through which PWID in Baltimore initiated drug use and transitioned to drug injection has evolved, correlating with an extremely high mortality rate particularly among those born after 1980.

Consistent with prior studies, we observed a trend towards increasing initiation with prescription drugs as opposed to more traditional pathways of non-injection cocaine and heroin use [37]. More than 40% of PWID born in the 1980s in both recruitment periods had initiated drug use with prescription drugs either alone or in combination with other illicit drugs, which was significantly higher than what was reported by participants born before 1980. While the estimates of the median age of initiation, years to injection drug use, and number of drugs used prior to injection initiation often varied by only a unit of one or two, they nonetheless provide important epidemiological insight. For example, PWID who initiated with prescription drugs faced similar or higher levels of drug-related harm as those who initiated through non-injection cocaine and heroin. Specifically, those who were born after 1980 tended to use more illicit substances prior to initiating injection drug use, which might be an important consideration when developing effective interventions to prevent injection initiation among young people who use drugs.

Overall, our data are consistent with recent findings from a multisite study of PWID in California [13] which demonstrated that persons born after 1980 had a shorter time to injection initiation. There was one exception; individuals in the 2015–2018 recruitment period who were born after 1980 and initiated drug use with prescription opioids and non-opioids tended to initiate injection after longer intervals compared to those who initiated drug use prior to 1980. One possible explanation for the longer time is the “two-step” process to injection initiation; [38] namely that prescription drug use precedes non-injection heroin use which precedes the transition to injection drug use [39]. Moreover, our results add to the literature by demonstrating that persons initiating with non-opioid prescription drugs had the longest time to initiation. While our data do not suggest that these individuals transitioned to prescription opioids before using/injecting heroin, there may be key differences between groups that initiate with prescription opioid vs non-opioid drug that will be important to designing interventions.

The longer time to injection initiation presents a window of opportunity to intervene and prevent the transition to injection of illicit opioids. Specifically, there are opportunities to ensure the availability and accessibility of effective drug treatment services, such as methadone and buprenorphine maintenance programs. Previous studies have shown that access to drug treatment can delay or prevent injection initiation[13, 40]. Social marketing campaigns and peer education interventions have also shown promise as potentially effective in preventing initiation [41, 42]. Furthermore, among those who already have initiated injection drugs, access to syringe exchange programs is needed in potentially underserved settings, such as suburban areas outside Baltimore City to prevent transmission of HIV and HCV. Safe injection facilities might also be critical in preventing injection initiation by limiting attendance only to PWID thereby reducing opportunities for PWID to initiate non-PWID to injection drug use [43].

Early intervention is also important to prevent the high rates of premature mortality that have been observed in this cohort and elsewhere [44, 45]. Since the late 1980’s, we have observed substantial declines in AIDS-related and drug-related mortality in this cohort commensurate with expansion of highly active antiretroviral therapy and harm reduction. However, over the past few years, drug-related mortality has increased in our cohort [46] consistent with national and local trends. Recent data suggest that approximately 30–50% of opioid related overdose deaths in Baltimore in 2015–2016 were attributed to fentanyl, [27] which is magnitudes more potent than heroin adulterated with non-synthetic opioid compounds [47]. Our data reinforce these national and local trends with the high SMRs particularly in PWID between the ages of 35–44. Indeed, according to 2017 data from the Maryland Department of Health, compared to the previous year, a 51% increase in the number of fentanyl related deaths was observed among individuals aged 35–44. Due the heightened risk of mortality, [48] it was further concerning to observe the heavier polysubstance use prior to injection initiation among PWID born after 1980.

### Limitations

This analysis is subject to several limitations. Firstly, we were limited to cross-sectional data of participants who had already initiated injection drug use by the time they enrolled in the study. As such, recall of exact ages of initiating drug use as well as the type of drug initiation may be imprecise. Indeed, a sizeable proportion of individuals had initiated prescription drugs and other illicit drugs during the same year, precluding us from establishing temporality. Because we wanted to avoid misclassification, we treated them as a separate group, however this grouping complicated direct comparisons between participants who initiated on prescription drugs and those who initiated on other illicit non-injection drugs. Secondly, we did not collect non-medical prescription drug use in the earlier cohorts (prior to 2005); thus we were unable to provide a more comprehensive analysis of the temporal patterns of drug use among PWID in Baltimore over a longer period. Thirdly, our samples may not be representative of the underlying PWID population since random sampling of hidden populations is not possible. However, our recruitment procedures were applied uniformly across both recruitment periods; thus the differences we observed in the 2005–2008 and 2015–2018 recruitment periods were most likely not due to how or from where the participants were recruited.

## Conclusions

These data suggest a changing profile of injection drug use in this urban setting consistent with shifts that have been observed across the U.S. Compared to those who were born before 1980, persons born after 1980 who initiated with either prescription opioids or non-opioids versus other non-injection illicit drugs, started illicit drug use at younger ages and reported higher levels of polysubstance use. Interventions will need to evolve to engage people who use drugs in harm reduction services early to prevent transitions into injection and associated consequences. Trends in mortality among PWID need to be continuously monitored given that injection drug use drives mortality through constantly changing and interrelated individual (e.g. polysubstance use) and structural level factors (e.g. incarceration). Among those with opioid use disorder, such interventions should consider strategies to increase access and retention while reducing economic and logistical barriers to methadone or buprenorphine maintenance treatment programs among young, non-injecting opioid users who are at high-risk of transitioning to injection drug use.

## Supporting information

S1 File Crude mortality rate by drug initiation type stratified by recruitment cohort. (DOCX)
