# Supplementary material for: Rising role of prescription drugs as a portal to injection drug use and associated mortality in Baltimore, Maryland
Source: PLoS One. 2019 Mar 4;14(3):e0213357. doi: 10.1371/journal.pone.0213357 (PMC6398863; doi:10.1371/journal.pone.0213357)

**S1 Fig. Crude mortality rate by drug initiation type stratified by recruitment cohort.** Black circles correspond to participants recruited from 2005 – 2008 and grey circles correspond to participants recruited from 2015 – 2018.


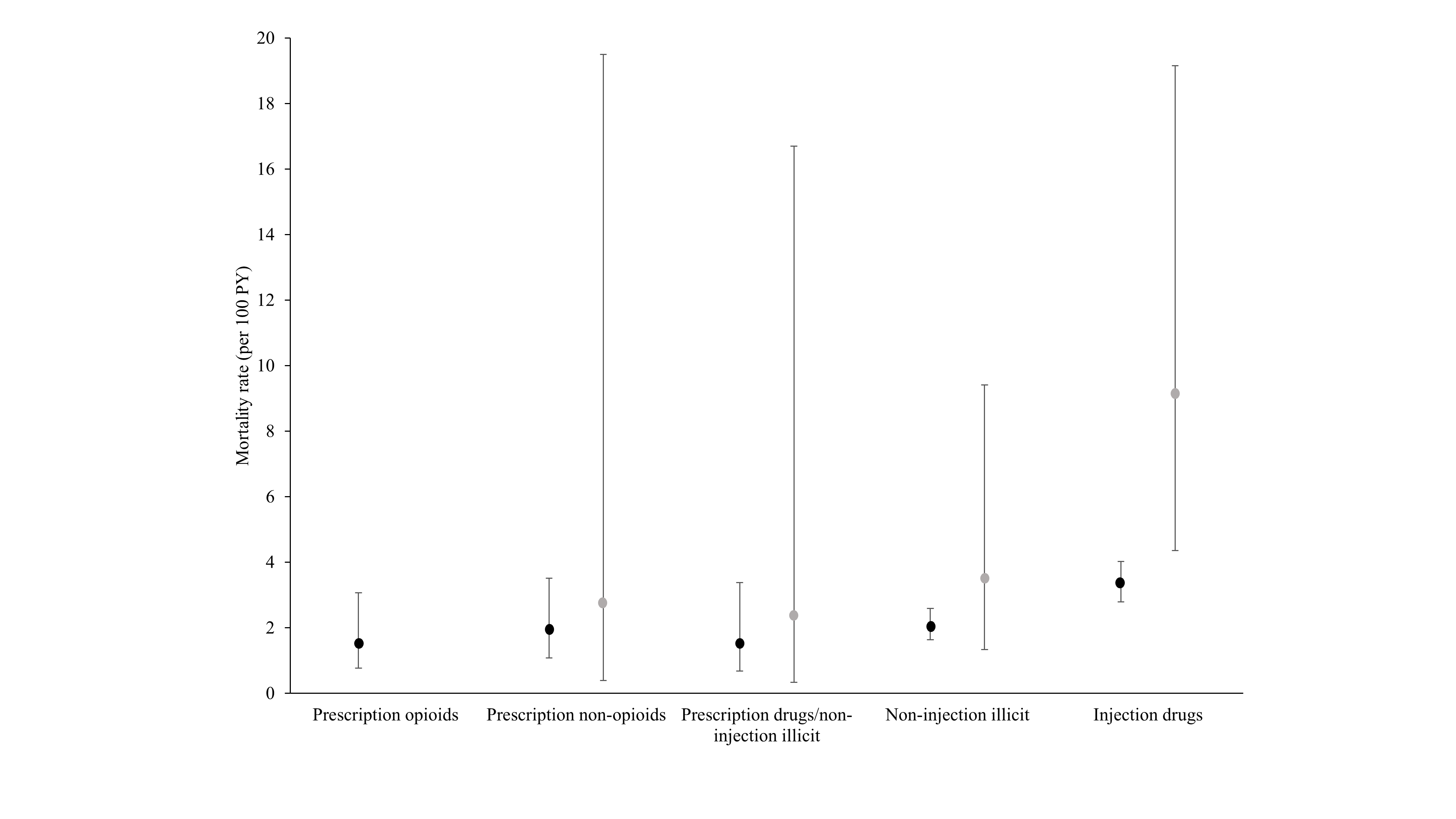

Supplement: S1 File — (DOCX) [file pone.0213357.s001.docx]
